# Properties of Composite Resins Early After Curing: Color Stability, Degree of Conversion, and Dimensional Stability

**DOI:** 10.1155/ijod/3702687

**Published:** 2025-07-30

**Authors:** Sahar Amirinejad, Narges Panahandeh, Hassan Torabzadeh

**Affiliations:** Department of Restorative Dentistry, Dental Research Center, Research Institute of Dental Sciences, School of Dentistry, Shahid Beheshti University of Medical Sciences, Tehran, Iran

**Keywords:** composite resins, Fourier transform infrared, polymerization, spectrophotometry, spectroscopy

## Abstract

**Objectives:** This study assessed the degree of conversion (DC), color, and dimensional stability, of three composite resins early after curing.

**Materials and Methods:** This study was conducted on X-tra fil, Filtek Z250, and Filtek Ultimate composites. To measure DC, the absorption spectra of composites before and after curing (immediately, 15 min, 2 h, 6 and 24 h, 1 week, and 1 month) were recorded by ATR-FTIR. Fifteen specimens were fabricated for each composite and assigned to two experimental groups (15 min immersion in coffee immediately and 2 h after curing) and a control. The CIE *L*^*⁣*^*∗*^^*a*^*⁣*^*∗*^^*b*^*⁣*^*∗*^^ color parameters of each specimen and color change (*∆E*) were measured at the aforementioned time points. Dimensional changes were measured by a digital micrometer. Respective ANOVA and Tukey tests were used for analysis. *p*  < 0.05 was considered significant.

**Results:** All composites exhibited an increased DC and expansion over time (*p*  < 0.001). At 1 month, the *∆E* of composites immersed in coffee after 2 h was not significantly from the control group. In the immediate coffee immersion group, X-tra fil experienced significantly greater *∆E* than Filtek Ultimate (*p* = 0.02). No other significant differences were found.

**Conclusion:** Time of exposure to coffee after curing had a significant effect on the color stability of X-tra fil only. X-tra fil showed minimum dimensional changes after 1 month.

## 1. Introduction

Composite resins are among the most commonly used tooth-colored restorative materials, which should have high color stability to maintain their color match over time and provide acceptable esthetic results [1]. In addition to secondary caries, color change is also a main cause of composite restoration replacement [2]. Color stability of composite restorations depends on their composition, quality of finishing and polishing of restoration, and the diet of the patient [3]. Dental materials are exposed to a dynamic oral environment, which can also affect their color stability. Discoloration of the polymer matrix is a common cause of color change in composite resins [4]. Resins with water sorption can also absorb other fluids, which results in their discoloration [1]. Composite resins with lower filler content have higher resin matrix volume, and subsequently greater water sorption and color change [5, 6]. Degree of conversion (DC) is another factor affecting the color stability of materials. The residual monomer in the polymer chain can lead to the formation of colored products and enhance the penetration of solvents and chromogenic substances from the oral environment into the restoration structure [1, 4].

External discoloration of composite resins with lower DC has been less commonly addressed in the literature, and the majority of available studies have focused on color stability of composite resins against internal discoloration in relation to their DC. Also, studies on discoloration of composite resins have mainly evaluated their discoloration over long periods of time, and their discoloration early after curing has been less commonly evaluated.

Given that the color stability of composite resins is correlated with their DC, and their polymerization is a continuous process, it remains uncertain whether the primary DC of composites after curing is sufficient to prevent their discoloration in short-term exposure (in the first day after curing) to chromogenic substances. Many dental clinicians request that patients refrain from consumption of coloring food and drinks in the first hours after receiving a tooth-colored restoration. Thus, it is imperative to assess the color stability of different types of composite resins early after curing. Additionally, the effect of time interval between curing and exposure to chromogenic substances on discoloration has not been previously evaluated.

Since, bulk-fill composites are a new generation of composite resins with a different composition, their color stability and DC have not been extensively studied. This study aimed to assess the color change and DC of three composite resins after exposure to coffee, as well as their dimensional stability in the first hours after curing. The first null hypothesis was that the DC of composite resins would not change over time. The second null hypothesis was that the tested composite resins would not be more susceptible to discoloration early after curing. The third null hypothesis was that the tested composite resins would not undergo dimensional changes over time.

## 2. Materials and Methods

This study was conducted using X-tra fil bulk-fill multi-hybrid composite resin (VOCO GmbH, Germany), Filtek Z250 microhybrid universal composite resin (3M ESPE, St. Paul, MN, USA), and Filtek Ultimate (3M ESPE, St. Paul, MN, USA) nanofilled universal composite resin.  Table 1 presents the properties of the three composite resins evaluated in this study.

### 2.1. Assessment of DC

DC of composite resins was measured by attenuated total reflectance-Fourier transform infrared spectroscopy (ATR-FTIR; Nicolet iS10, ThermoFisher Scientific) using Omnic Spectra software. For this purpose, first, the absorption spectra of each composite paste were measured. Subsequently, three disc-shaped specimens were fabricated from each composite resin using a stainless steel mold measuring 8 mm x 2 mm. Composite resins were applied in the mold on a glass slab with a celluloid matrix band over it. The composite surface was also covered with another celluloid matrix band, and a glass slab was placed over it and compressed with hand pressure to achieve a uniform thickness and a smooth surface. The glass slab was then removed and each specimen was cured according to the manufacturers' instructions. Accordingly, X-tra fil composite specimens were cured for 10 s, while Filtek Z250 and Filtek Ultimate were cured for 20 s by a LED curing unit (Guilin Woodpecker Medical Instrument Co., China) with an intensity of 950 mW/cm^2^ through the celluloid matrix band. The light intensity was checked by a LED radiometer (SDI) prior to curing of each specimen. The specimens were then removed from the mold, and excess material was removed from the edges. The absorption spectra of each specimen were measured immediately after curing and also after 15 min, 2 h and 15 min, 6 and 24 h, 1 week, and 1 month at the center of their upper surface. The absorption spectra were recorded by 16 scans with 4 cm^−1^ resolution at 400–4000 cm^−1^ wavelength range using ATR-FTIR (Nicolet iS10, ThermoFisher Scientific). The specimens were stored in 20 mL of distilled water and incubated at 37°C between the measurements, and dried with absorbent paper prior to each measurement.

To calculate the DC of each composite specimen at each time point, the stretching vibration peaks of the C═C aliphatic bonds (1637 or 1638 cm^−1^) were selected as the analytical absorption band. The stretching vibration peaks of the C─C aromatic bonds (1608 cm^−1^) that remain intact during the polymerization reaction were selected as the reference. To quantify the DC, the following formula was used:

  $$\begin{array}{c} \text{DC\% }1-\frac{\;\left(C=C/\text{aromatic}\right)\text{cured}}{\;\left(C=C/\text{aromatic}\right)\text{uncured}}\times 100. \end{array}$$

### 2.2. Assessment of Color Stability

Fifteen specimens were fabricated from each composite type as explained earlier. Five specimens from each composite type were assigned to the control group. The CIE *L*^*⁣*^*∗*^^*a*^*⁣*^*∗*^^*b*^*⁣*^*∗*^^ color parameters were measured by a spectrophotometer (Ci62, X-Rite, Grandville, MI, USA). The specimens were placed against a neutral standard white background (Color Checker Passport; X-Rite, Grandville, MI, USA), and the 4 mm diameter of the device was adapted to the center of each specimen surface. The measurements were made in the visible light spectrum at a 12° observer angle under the D65 illuminant. Each measurement was repeated three times for each specimen, and the mean of the three measurements was reported as the final value.

The control specimens were immersed in 20 mL of distilled water and incubated at 37°C. After 15 min, they were removed from the incubator, dried with a sterile gauze, and their color parameters were measured again by a spectrophotometer. They were then immersed again in distilled water and incubated. The color parameters of the specimens were measured after 2 h and 15 min, 6 and 24 h, 1 week, and 1 month after curing. The remaining 10 specimens fabricated from each composite type were assigned to two experimental groups for immersion in coffee solution. In the first experimental group, the specimens were immersed in 20 mL of coffee solution at 37°C after primary colorimetry, and incubated. After 15 min (which is the time it normally takes to drink a cup of coffee), the specimens were removed from the incubator and rinsed under distilled water for 60 s. They were then dried with sterile gauze, and their color parameters were measured again by a spectrophotometer. Next, each specimen was immersed in 20 mL of distilled water, and incubated. The specimens were removed after 2 h and 15 min, dried, and underwent colorimetry. They were then placed back in distilled water and incubated. The same process was repeated at 6 and 24 h, 1 week, and 1 month after curing.

In the second experimental group, the specimens were immersed in 20 mL of distilled water and incubated at 37°C. After 15 min, they were removed from the incubator, dried with sterile gauze, and their color parameters were measured. They were immersed again in distilled water and incubated. After 2 h, they were removed from the incubator, dried, and their color parameters were measured. Next, they were immersed in coffee solution at 37°C and incubated for 15 min. After 15 min (i.e., 2 h and 15 min after curing), they were removed from the solution, rinsed with distilled water for 60 s, and dried with sterile gauze. Their color parameters were then measured. The specimens were immersed again in distilled water and the same process was repeated after 6 and 24 h, 1 week, and 1 month after curing.

To prepare the coffee solution, 3.6 g of coffee powder (Nescafe Gold Blend, Nestle Global, Switzerland) was added to 400 mL of boiling water according to the manufacturer's instructions, the solution was stirred for 10 min while boiling, and was subsequently filtered using a coffee filter paper, resulting in a coffee solution with a concentration of 0.009 g/mL.

The *L*^*⁣*^*∗*^^, *a*^*⁣*^*∗*^^, and *b*^*⁣*^*∗*^^ color parameters were recorded by the Color iQC version 9.5.10 software. Color change (*∆E*) of each specimen was calculated after each measurement by comparing it with the baseline value (measured immediately after curing) using the following formula:

  $$\begin{array}{c} ∆{E^{{\;}^{*}}}_{ab}\;={\left(∆L^{{\;}^{*}}{\;}^{2}\;+∆a^{{\;}^{*}}{\;}^{2}\;+∆b^{{\;}^{*}}{\;}^{2}\right)}^{*1/2}. \end{array}$$

### 2.3. Assessment of Dimensional Changes

The specimens used for colorimetry were also used for the assessment of dimensional stability of composite resins. Linear dimensional changes were measured for this purpose, such that the thickness of each of the 45 specimens was measured by a digital micrometer (Mitutoyo, Japan) with 1 µm accuracy immediately after curing, and also after 15 min, 2 h and 15 min, 6 and 24 h, 1 week, and 1 month after measuring their color parameters. The micrometer was calibrated after each measurement. Dimensional changes of each specimen were calculated by subtracting the final thickness from the primary thickness divided by the primary thickness.

### 2.4. Statistical Analysis

Normal distribution of data was evaluated by one-sample Kolmogorov–Smirnov test. Considering the normal distribution of data (*p*  > 0.05), two-way repeated measures ANOVA was applied to compare the DC of different composite resins at different time points. One-way ANOVA with Tukey's test and one-way repeated measures ANOVA followed by the Bonferroni test were used to compare the DC, color change, and dimensional changes of composite resins at different time points. Three-way repeated measures ANOVA was applied to compare the color change and dimensional changes of different composite resins at different time points and different conditions. Two-way ANOVA was used to assess the effect of immersion in coffee on color parameters. All statistical analyses were carried out using SPSS 18 at a 0.05 level of significance.

## 3. Results

Table 2 presents the DC of different composite resins at different time points. The interaction effect of the time of assessment and type of composite on DC was significant (*p* = 0.024). In other words, the pattern of change in DC was not the same among different composites over time. Due to the presence of a significant interaction effect, a subgroup analysis was performed.

### 3.1. Comparison of DC of Different Composites at Each Time Point

One-way ANOVA was used to compare the mean DC of different composites at each time point. The results showed no significant difference at any time point among the materials (*p*  > 0.05) except for 2 h and 15 min (*p* = 0.007) and 6 h (*p* = 0.003). Pairwise comparisons of the groups at the above-mentioned two time points by the Tukey's HSD test showed that at 2 h and 15 min, Filtek Ultimate had a significantly lower mean DC than X-tra fil (*p* = 0.006). No other significant differences were found (*p*  > 0.05). At 6 h, Filtek Z250 had significantly higher DC than Filtek Ultimate (*p* = 0.043), and X-tra fil had significantly higher DC than Filtek Ultimate (*p* = 0.003).

### 3.2. Comparison of the DC of Each Composite Over Time (at Different Time Points)

One-way repeated measures ANOVA showed a significant increase in the DC of each composite over time (*p*  < 0.001 for all three). Pairwise comparisons of the time points by the Bonferroni test showed higher DC of X-tra fil at 1 week compared with immediately after curing (*p* = 0.015). No other significant differences were noted for X-tra fil. The DC of Filtek Z250 at 1 week and 1 month was significantly higher than that of immediately after curing (*p*  < 0.05). No other significant differences were noted for Filtek Z250 (*p*  > 0.05). The DC of Filtek Ultimate at 1 month after curing was higher than that of the 2 h and 15 min (*p* = 0.012). Also, its DC at 1 week was higher than that of the 6 h after curing (*p* = 0.041). No other significant differences were noted for Filtek Ultimate (*p*  > 0.05).

### 3.3. Color Stability

Table 3 presents the *∆E* of the groups. Two-way ANOVA showed a different effect of coffee exposure on different composites immediately after exposure (*p*  < 0.001). Also, the time interval between curing and coffee exposure had a significant effect on different composites (*p*  < 0.05). However, the interaction effect of the type of composite and time interval between curing and coffee exposure was not significant (*p* = 0.07). Pairwise comparisons of composite resins by the Tukey's HSD test showed that Filtek Ultimate and X-tra fil had maximum *∆E* with no significant difference between them (*p* = 0.409). However, Filtek Z250 had a lower *∆E* than X-tra fil (*p*  < 0.001) and Filtek Ultimate (*p* = 0.005). Comparison of different conditions revealed that the group immersed in coffee after 2 h had maximum *∆E*, while the control group had minimum *∆E*. All three comparisons yielded significant differences (*p*  < 0.05).

### 3.4. Comparison of the ∆E of Different Composites Under Different Conditions Over Time

Table 4 presents the *∆E* of different composites under different conditions over time. Three-way repeated measures ANOVA showed a significant interaction effect among the type of composite, condition, and time on *∆E*. Thus, subgroup analyses were performed.

### 3.5. Comparison of ∆E of Different Composites at Each Time Point Following Immersion in Distilled Water (Control Conditions)

One-way ANOVA showed a significant difference among different composites at 15 min (*p* = 0.049), 2 h and 15 min (*p* = 0.008), and 1 week (*p*  < 0.001) regarding *∆E*. No other significant differences were noted (*p*  > 0.05).

Pairwise comparisons by Tukey's test showed that at 15 min, Filtek Z250 had a significantly lower *∆E* than X-tra fil (*p* = 0.047). At 2 h and 15 min, Filtek Ultimate had a significantly lower *∆E* than X-tra fil (*p* = 0.008) and Filtek Z250 (*p* = 0.046). At 1 week, Filtek Z250 showed lower *∆E* than X-tra fil (*p*  < 0.001) and Filtek Ultimate (*p* = 0.003).

### 3.6. Comparison of ∆E of Different Composites Immersed in Coffee 2 h After Curing at Each Time Point

One-way ANOVA showed a significant difference among different composites regarding *∆E* at 2 h and 15 min (*p* = 0.031) and 6 h (*p* = 0.026). No other significant differences were noted (*p*  > 0.05). Pairwise comparisons by the Tukey's HSD test showed that at 2 h and 15 min, Filtek Ultimate had significantly higher *∆E* than Filtek Z250 (*p* = 0.033). Also, at 6 h, Filtek Ultimate had higher color change than X-tra Fil (*p* = 0.032).

### 3.7. Comparison of ∆E of Different Composites Immersed in Coffee Immediately After Curing at Each Time Point

One-way ANOVA showed a significant difference among different composites regarding *∆E* only at 1 month (*p* = 0.026). Pairwise comparisons by the Bonferroni test showed higher *∆E* of X-tra fil than Filtek Ultimate (*p* = 0.026).

### 3.8. Comparison of ∆E of X-tra Fil Specimens Immersed in Distilled Water and Coffee Immediately and 2 h After Curing at Different Time Points

One-way ANOVA showed a significant difference in *∆E* of X-tra fil specimens immersed in distilled water and coffee immediately and 2 h after curing at 15 min (*p*  < 0.001), 2 h and 15 min (*p* = 0.004), 6 h (*p* = 0.006), 1 week (*p*  < 0.001), and 1 month (*p* = 0.001). Pairwise comparisons at 15 min revealed significant differences between all three testing conditions, such that the minimum *∆E* was noted in immersion after 2 h, while the maximum *∆E* was recorded in the immediate immersion group. At 2 h and 15 min, pairwise comparisons revealed minimum *∆E* in the control group which had significant differences with immersion after 2 h (*p* = 0.005) and immediate immersion (*p* = 0.018) groups. At 6 h, pairwise comparisons showed that the control group had a significantly lower *∆E* than immediate immersion group (*p* = 0.004). The same results were obtained at 1 week and 1 month.

### 3.9. Comparison of ∆E of Filtek Z250 Specimens Immersed in Distilled Water and Coffee Immediately and 2 h After Curing at Different Time Points

One-way ANOVA revealed no significant differences in *∆E* of Filtek Z250 at any time point among the three testing conditions (*p*  > 0.05).

### 3.10. Comparison of ∆E of Filtek Ultimate Specimens Immersed in Distilled Water and Coffee Immediately and 2 h After Curing at Different Time Points

One-way ANOVA showed significant differences at all time points (*p*  < 0.05), except at 15 min (*p* = 0.219). The Tukey's HSD test showed that at 2 h and 15 min, *∆E* was significantly higher in the immersion after 2 h group compared with the immediate immersion group (*p* = 0.019) and the control group (*p* = 0.001). The same results were obtained at other time points.

## 4. Dimensional Stability

### 4.1. Comparison of Dimensional Stability of Different Composites Over Time

Table 5 presents the percentage of dimensional changes of composite specimens over time.

Two-way repeated measures ANOVA showed significant interaction effect of type of material and time on dimensional stability (*p*  < 0.001). Thus, subgroup analyses were performed.

### 4.2. Comparison of Dimensional Stability of Different Composites at Each Time Point

One-way ANOVA showed a significant difference in dimensional stability of different composite resins only at 1 month (*p*  < 0.05). Pairwise comparisons by the Bonferroni test showed that Filtek Z250 and Filtek Ultimate had the maximum percentage of dimensional change with no significant difference (*p* = 0.334). X-tra fil had significantly lower mean percentage of dimensional change (*p*  < 0.05).

### 4.3. Comparison of Dimensional Changes of Each Composite Over Time

One-way repeated measures ANOVA showed a significant change in dimensional stability of all three composites over time (*p*  < 0.001 for all three). Pairwise comparisons of the time points showed that in X-tra fil, the percentage of dimensional change at 2 h and 15 min was lower than that of 1 month (*p* = 0.008). Also, at each time point, the percentage of dimensional change was significantly lower than that at later time points (*p*  < 0.05).

In Filtek Z250, the percentage of dimensional changes at 15 min was lower than that of 1 week and 1 month (*p*  < 0.05). The same results were obtained at 2 h and 15 min, 6 and 24 h. At 1 week, the percentage of dimensional changes was lower than that of 1 month (*p*  < 0.001).

In Filtek Ultimate, the percentage of dimensional changes at 15 min, 2 h and 15 min, 6 and 24 h was significantly lower than that of 1 week and 1 month (*p*  < 0.001 for all). The percentage of dimensional changes at 1 week was also lower than that of 1 month (*p* = 0.001).

## 5. Discussion

This study aimed to assess the color change and DC of three composite resins after exposure to coffee and their dimensional stability early after curing. Evaluation of DC and dimensional stability at the same time point was done to assess the possible correlation between these properties. The first null hypothesis was that DC of composite resins would not change over time. The second null hypothesis was that the tested composite resins would not be more susceptible to discoloration early after curing. The third null hypothesis was that the tested composite resins would not undergo dimensional changes over time.

### 5.1. DC

Evidence shows that DC of composite resins is significantly correlated with their strength and coefficient of elasticity [7]. Low DC and high percentage of residual monomers can lead to lower biocompatibility, discoloration of the restoration, increased wear, and poor mechanical properties [8]. An increase in DC of composite resins over time (within the first 24 h) was reported by Alshali et al. [7], although the trend of increase was different depending on the chemical composition of composite resins. The same result was reported by Al-Ahdal et al. [9], who assessed the DC of composite resins at 5, 30, and 60 min and 24 h. However, maximum DC of different composites was recorded at different times.

In the present study, all three composites showed an increase in their DC over time. Thus, the first null hypothesis was rejected. Although the trend of increase was slightly different among them, the maximum DC in all three composite resins was noted at 1 week after curing, and no further increase occurred at 1 month. At both immediately after curing and after 1 month, X-tra fil showed maximum and Filtek Ultimate showed minimum DC, although the differences did not reach statistical significance. Lack of a significant difference among the three composites in this respect can be due to their almost similar resin matrix composition. Monomers affect the DC through their viscosity. X-tra fil has bis-GMA (bisphenol A-glycidyl methacrylate), UDMA (urethane dimethacrylate), TEGDMA (triethylene glycol dimethacrylate), MMA, and bis-EMA in its resin matrix. Bis-GMA is the most viscous monomer; however, the addition of TEGDMA plasticizes the network and increases the speed of polymerization and DC. Bis-EMA is also a heavy monomer with a hard phenyl ring, which decreases the DC and mobility of molecules, such as UDMA. UDMA has a much lower viscosity than bis-GMA, and has greater mobility. The imino group in UDMA enhances the continuation of polymerization. The resin matrix of Filtek Z250 and Filtek Ultimate is composed of monomers similar to those in X-tra fil, and contains bis-GMA, UDMA, TEGDMA, and bis-EMA. Their manufacturer claims that TEGDMA has been mainly replaced with a mixture of UDMA and bis-EMA. In composites based on bis-GMA monomer, the most important factor affecting the continuation of polymerization after light curing is the amount of TEGDMA. UDMA also has low viscosity and high potential for continuation of polymerization reactions while bis-EMA, which has replaced part of TEGDMA, is a heavy viscous molecule and prevents the mobility of UDMA. Thus, the difference in DC of these two composites with that of X-tra fil is probably attributed to the reduction of TEGDMA and its replacement with the abovementioned monomers [7, 10–13]. The mineral content of composite resins also affects their polymerization. A previous study reported a reduction in DC of composites by an increase in their mineral content [14]. Al-Ahdal et al. [9] reported that increased filler content decreased postcuring polymerization. X-tra fil, which showed higher DC in the present study, has a higher filler content (70.1%) than Filtek Z250 (60%) and Filtek Ultimate (63.3%). This finding was in contrast to the results of the aforementioned studies, which may be due to differences in resin matrix composition. On the other hand, Amirouche-Korichi et al. [12] showed that fillers had no significant effect on the degree of mobility of matrix molecules, and affected the DC through their size and surface area. Dionysopoulos et al. [13] evaluated the effect of composite type on the efficacy of curing and showed that X-tra fil had maximum hardness at both 2 and 4 mm depths. They attributed this finding to the large size of fillers (>20 µm) which increases the translucency. Thus, higher filler percentage in X-tra fil may increase the translucency and subsequent passage of light and eventually increase the DC. Although, the photo-initiator of all three composites is based on camphorquinone (CQ), incorporation of a new photo-initiator in the composition of X-tra fil as claimed by the manufacturer may be responsible for its higher DC. Slight difference in the DC of Filtek Ultimate and Filtek Z250 may be due to the presence of nanofillers and nanoclusters in the composition of Filtek Ultimate, which affect light reflection and diffraction, and decrease its DC compared with that of Filtek Z250. Lower DC of nanofilled compared with microhybrid composites has also been reported in previous studies [15–17].

### 5.2. Color Stability

The present results showed no significant difference in color change of different composites in the control group and the group immersed in coffee 2 h after curing at 1 month. However, X-tra fil bulk-fill composite in the immediate immersion group experienced greater color change than the conventional composites. The results indicated that the time of exposure to a chromogenic substances after curing affects the color change, and X-tra fil experienced greater color change in all conditions. Thus, the second null hypothesis was also rejected. This finding may be due to the more hydrophilic resin matrix of X-tra fil and its higher susceptibility to color change. As mentioned earlier, the presence of higher amounts of TEGDMA in its resin matrix is probably responsible for its greater color change. Lower color stability of Tetric EvoCeram bulk-fill composite in coffee solution was also reported by Shamszadeh et al. [18] Moreover, Toz Akalin et al. [19] reported higher color change of SonicFill bulk-fill composite in several mouthwashes. However, different results were reported by Barakah and Taher [20] and Koc-Vural et al. [21], who reported lower color change of Tetric EvoCeram bulk-fill compared with Filtek Ultimate nanofilled composite, despite the rougher surface of the bulk-fill composite after polishing. They attributed the higher color change of the nanofilled composite to its more hydrophilic matrix, and the presence of TEGDMA monomer in its composition [20, 21]. According to the manufacturer, Tetric EvoCeram contains bis-GMA, bis-EMA, and UDMA monomers in its composition, and the lack of TEGDMA may explain its higher color stability, while X-tra fil contains TEGDMA in its resin matrix, which is probably responsible for its greater color change.

Controversy exists regarding the color stability of nanofilled and microhybrid composite resins [22, 23]. In the present study, no significant difference was noted in color stability between nanofilled and microhybrid composite resins, which is probably due to the similarity of their resin matrix. The present results did not show a correlation between DC and color stability of different composite resins, since the three composites had no significant difference regarding DC at 1 month, while a significant difference in color change was noted in the immediate immersion group. This result was in line with the findings of Sarafianou et al. [24], and Al-Kheraif [2], who found no correlation between DC of different materials and their color change. However, this finding was in contrast to the results of Aguiar et al. [25], since they found a direct correlation between DC and color change of composite resins. This controversy may be due to the long exposure time of specimens to coffee (40 min a day for 40 days) in their study, causing greater color change of composite resins. Imazato et al. [26], also pointed to a direct correlation between DC and color change of composite resins. However, they evaluated color change after long-term aging of specimens, which was different from our methodology. In the present study, *∆E* >3.3 was considered clinically unacceptable [6]. Accordingly, clinically unacceptable color change did not occur in any specimen at 1 month in the present study except for Filtek Ultimate specimens immersed in coffee 2 h after curing at 2 h and 15 min and 6 h. The color change of X-tra fil was visually perceivable but within the clinically acceptable range.

It is worth noting that nowadays *Δ*E00 (CIEDE2000) is a more perceptually uniform method and is currently preferred for dental applications and should be considered as a new method for assessing color change for future research.

### 5.3. Dimensional Stability

Dimensional changes of composite resins are the result of their negative dimensional change due to polymerization shrinkage and positive dimensional change due to water sorption. Water sorption negatively affects the mechanical properties, causes discoloration, decreases the wear resistance, and results in hydrolytic degradation of the resin-filler interface [14]. Bulk-fill composites have a plasticizer in their resin matrix which may change their resistance to water sorption [14]. The present results revealed a significant increase in volume of all three composite types over time. Thus, the third null hypothesis was rejected as well. This finding was in line with the results of Gusmão et al. [27], reporting an increase in water sorption of composite resins over time. At the final time point, X-tra fil showed significantly lower dimensional changes than the other two, while Filtek Ultimate and Filtek Z250 were not significantly different in this respect. This finding was unexpected considering the presence of higher amounts of hydrophilic TEGDMA in its composition. It should be noted that X-tra fil experienced negative dimensional changes up to 6 h after curing (which did not occur in such a high intensity in the other two composites); this may explain lower dimensional changes at the end. Wei et al. [28] assessed the correlation of hygroscopic dimensional changes and water sorption and reported that this correlation was not linear, particularly in the first hours. This can lead to occupation of micro-voids in composite resin by water molecules and water sorption without volumetric expansion. Considering the larger size of fillers in X-tra fil and probably higher microscopic porosities, lower volumetric expansion may not indicate lower water sorption in this composite.

Concerning the in vitro design of this study, generalization of the results to the clinical setting must be done with caution. Given that the manufacturer did not support the same color as other composites (i.e., A2) in its product portfolio, the closest color was used in this study. Obviously, due to the differences in optical behavior and pigment content, which may influence translucency, and light scattering, these optical properties can influence both the DC (via light penetration) and perceived color change (*ΔE*). This may partially explain X-tra fil's differing behavior. However, it should be emphasized that the intragroup comparisons are possible. The above-mentioned point can be considered as a limitation of this study.

## 6. Conclusion

The DC of all three composites increased over time and reached its maximum value after 1 week. The three tested composites had no significant difference regarding their maximum DC. X-tra fil experienced significantly greater color change after 1 month in the immediate immersion group. Also, time of exposure to coffee after curing was an important factor that affected the color stability of X-tra fil, unlike the other two composites. Although all specimens showed perceivable color change, clinically unacceptable color change did not occur in any group. Moreover, all composites experienced dimensional changes after 1 month but dimensional changes in X-tra fil were significantly lower compared with the other two composites.

## Figures and Tables

**Table 1:** Properties of the three composite resins evaluated in this study.

Composite type	Manufacturer	Color	Filler content	Batch number	Resin matrix components	Photoinitiators
X-tra fil	VOCO GmbH, Anton-Flettner-Str. 1–3, 27472, Cuxhaven, Germany	Universal	86% weight 70.1% volume	1628099	Bis-GMA (bisphenol A-glycidyl methacrylate) TEGDMA (triethylene glycol dimethacrylate) UDMA (urethane dimethacrylate)	• Camphorquinone (CQ) and broad-wavelength LED compatible resin (not stated exactly with manufacturer)

Filtek Z250	3M ESPE Dental Products 2510 Conway Avenue St. Paul, MN 55144–1000 USA	*A*_2_	82% weight 60% volume	N737300 N736817	Bis-GMA UDMA Bis-EMA (bisphenol A-polyethylene glycol diether dimethacrylate)	• CQ

Filtek Ultimate	3M ESPE Dental Products 2510 Conway Avenue St. Paul,MN 55144–1000 USA	Dentin *A*_2_	78.5% weight 63.3% volume	N730696 N734592	Bis-GMA, UDMA, TEGDMA, Bis-EMA, PEGDMA (polyethylene glycol dimethacrylate)	• CQ and tertiary amines

**Table 2:** DC of different composite resins at different time points.

Composite type	Degree of conversion (%) (mean ± SD)						
	Immediately	15 min	2 h and 15 min	6 h	24 h	1 week	1 month
X-tra fil	2.31 ± 62.23	2.52 ± 65.33	1.53 ± 6.33	1.73 ± 67.00	2.00 ± 8.00	2.65 ± 75.00	2.08 ± 74.33
Z250	3.61 ± 61.00	3.46 ± 62.00	2.65 ± 62.00	2.08 ± 63.33	1.15 ± 66.33	3.21 ± 72.67	2.65 ± 73.00
Ultimate	2.31 ± 56.67	1.53 ± 58.67	2.08 ± 57.67	1.00 ± 59.00	1.00 ± 68.00	0.00 ± 72.00	1.53 ± 71.33

*Note*: Three specimens in each group.

**Table 3:** *∆E* of the groups.

Composite type	Group	*∆E* (mean ± SD)
X-tra fil	Control	1.37 ± 0.83
	2 h	3.02 ± 0.58
	Immediately	2.86 ± 0.19

Z250	Control	0.36 ± 0.25
	2 h	1.46 ± 0.76
	Immediately	1.18 ± 0.39

Ultimate	Control	0.63 ± 0.54
	2 h	3.64 ± 1.15
	Immediately	1.82 ± 1.64

*Note*: Five specimens in each group.

**Table 4:** *∆E* of different composites under different conditions over time.

Composite	Group	*∆E* at 15 min (mean ± SD)	*∆E* at 2 h and 15 min (mean ± SD)	*∆E* at 6 h (mean ± SD)	*∆E* at 24 h (mean ± SD)	*∆E* at 1 week (mean ± SD)	*∆E* at 1 month (mean ± SD)
X-tra fil	Control	1.37 ± 0.83	1.21 ± 0.44	0.99 ± 0.62	1.09 ± 0.89	0.87 ± 0.14	1.09 ± 0.51
	2 h immersion	0.26 ± 0.25	2.34 ± 0.60	1.69 ± 0.32	1.89 ± 0.72	2.61 ± 0.34	1.98 ± 0.19
	Immediate immersion	2.86 ± 0.19	2.15 ± 0.25	2.13 ± 0.33	1.93 ± 0.23	2.34 ± 0.20	2.13 ± 0.28

Z250	Control	0.36 ± 0.25	0.98 ± 0.46	1.12 ± 0.54	1.76 ± 0.42	1.81 ± 0.36	1.51 ± 0.34
	2 h immersion	0.73 ± 0.76	1.90 ± 0.98	1.96 ± 0.85	2.30 ± 0.90	2.17 ± 0.93	1.81 ± 0.88
	Immediate immersion	1.18 ± 0.39	1.32 ± 0.40	1.64 ± 0.42	2.28 ± 0.39	2.17 ± 0.42	1.50 ± 0.28

Ultimate	Control	0.63 ± 0.54	0.33 ± 0.14	0.52 ± 0.20	0.97 ± 0.08	1.21 ± 0.12	1.40 ± 0.27
	2 h immersion	0.85 ± 0.69	3.92 ± 1.52	3.66 ± 1.61	3.28 ± 1.46	3.20 ± 1.22	2.71 ± 0.87
	Immediate immersion	1.82 ± 1.64	1.48 ± 1.41	1.56 ± 1.23	1.53 ± 1.02	1.34 ± 0.95	1.28 ± 0.65

*Note*: Five specimens in each group.

**Table 5:** Percentage of dimensional changes of composite specimens over time.

Composite type	Dimensional change (%) (Mean ± SD)					
	15 min	2 h and 15 min	6 h	24 h	1 week	1 month
X-tra fil	0.01 ± 0.04	−0.02 ± 0.05	−0.03 ± 0.09	±0.09	0.04 ± 0.08	0.07 ± 0.09
Z250	±0.02	0.01 ± 0.05	0.02 ± 0.06	0.04 ± 0.07	0.09 ± 0.07	0.16 ± 0.06
Ultimate	±0.03	−0.01 ± 0.07	0.01 ± 0.06	0.02 ± 0.07	0.07 ± 0.06	0.21 ± 0.01

*Note*: Fifteen specimens in each group.

## Data Availability

Data will be available upon request from the authors.
